# Associated factors with the knowledge of nurses of a high complexity oncology centre in Brazil, on the management of cancer pain

**DOI:** 10.3332/ecancer.2019.928

**Published:** 2019-05-09

**Authors:** Flávia dos Santos Ferreira, Karina Cardoso Meira, Rayane Saraiva Félix, Iara Rayane Silva de Oliveira, Cecilia Maria Izidoro Pinto, Magda Aparecida dos Santos Silva, Juliano dos Santos

**Affiliations:** 1 Florence Clinic: Rehabilitation and Palliative Care, Bela Vista do Cabral Street, 271, Nazaré, BA, Brazil; 2 Health School Rio Grande do Norte Federal University, Senador Salgado Filho, Avenue, s/n Lagoa Nova, Natal, RN, Brazil; 3 Medical-surgical Nursing Department, Anna Néry Nursing School/UFRJ, Afonso Cavalcanti Street, 275-Cidade Nova, Rio de Janeiro, RJ, Brazil; 4 Nursing Department of Paulista University- Jacery Street, 247-Morumbi, São Paulo, SP, Brazil; 5 José de Alencar Gomes da Silva National Institute of Cancer-INCA Cruz Vermelha Square, 23-Centro, Rio de Janeiro, RJ, Brazil; a https://orcid.org/0000-0002-6022-8803; b https://orcid.org/0000-0003-2025-8642; c https://orcid.org/0000-0001-5490-0803; d https://orcid.org/0000-0003-2433-2811; e https://orcid.org/0000-0002-1722-5703; f https://orcid.org/0000-0001-9961-3576

**Keywords:** oncology nursing, pain management, nursing care, nursing education, teaching

## Abstract

Pain is one of the most prevalent symptoms in cancer patients and may be directly related to cancer or to the procedures needed for its diagnosis and treatment. It is estimated that about 40% of cancer patients receive inadequate treatment for painful conditions. Among the barriers to adequate pain management are inadequate knowledge and the dysfunctional beliefs of healthcare professionals. Therefore, the present study aims to assess the knowledge of oncology nurses on the management of pain, as well as the factors associated with it. It is a cross-sectional study with 126 nurses working at a High Complexity Oncology Centre in Brazil. Knowledge about the management of cancer pain was evaluated through the instrument ‘Nurses’ Knowledge on Cancer Pain Management—World Health Organization—developed by Ramos (1994). In the analysis of the association between knowledge about pain management and the independent variables, Poisson regression was used with robust variance, and values of p ≤ 0.05 were considered statistically significant. Adequate knowledge prevalence was 54.1% confidence intervals (CI 5.40%–62.80%). These nurses differed in relation to those with inadequate knowledge regarding the source of knowledge about pain, the ethical aspects in the treatment of the patient with oncologic pain, and non-pharmacological methods (coeliac plexus neuroleptic block) for pain control. Also, the factors associated with adequate knowledge were longer professional experience time ([10–19 years (ratio prevalence (RP) = 1.72, 95% CI: 1.05–2.81), 20–29 years (RP = 2.56, 95% CI: 1.63–4.02), 30–39 years (RP = 3.45, 95% CI: 2.25–5.29]), and not believing that the use of opioids causes harm to patients corresponded with a greater chance prevalence ratio (PR = 1.20, 95% CI: 1.12–1.20) of having adequate knowledge. The findings of the study point to the need for continuing education, updated education, and reflection, especially for nurses with less professional experience.

## Introduction

Cancer pain can be defined as ‘total pain’, representing a syndrome that goes beyond nociception (injury), also involving physical, emotional, social and spiritual factors [1]. It is estimated that in recent decades this symptom has affected around 17 million people in the world, and its prevalence increases progressively with the disease, ranging from 39.3% of patients reporting pain during curative treatment to 66.4% in metastatic disease or end-of-life patients [2].

The painful condition may be directly related to the tumour (bone, nerve and/or soft tissue compression), muscle spasms, lymphedema, pressure ulcers, intestinal constipation, and the procedures necessary for diagnosis and treatment [1, 2], being potentiated by fear, anxiety and depression [2]. Locations that present pain in more than 50% of the affected patients are: head and neck (70%); gynaecological (60%); gastrointestinal (59%); lung (55%); breast (54%) and urogenital cancer (52%) [2].

It is estimated that about 43% of people living with cancer receive inadequate treatment for their pain, an unacceptable reality considering that since 1986, there is a treatment protocol advocated by the World Health Organization (WHO), which promotes pain control in 70%–90% of patients, when applied correctly [2, 3].

Studies advocate that barriers to the proper management of painful conditions in cancer patients are due to lack of knowledge and dysfunctional beliefs about pain and analgesia related to patients, family members and health professionals [2, 4–6].

Such realities may contribute to the undertreatment of pain, directly influencing the quality of life of those in a painful condition, also, the persistence and non-relieving of the pain complaint can lead to a lack of trust between the patient/family and the assistant team furthering nonadherence to the proposed treatment [3].

Research has pointed out that the inadequate management of therapeutic practices by health professionals is associated with a lack of educational programmes, absence of pre-established routines, fear of addiction and adverse reactions of opioid-based drugs, lack of knowledge of methods for assessing pain and inadequate beliefs about pain [1, 3, 4, 7–14] representing a barrier to adequate pain control and resulting in discomfort and physical and emotional deterioration of the patient [15].

Facing this reality, and considering that pain is an important concern among cancer patients [16] and that for the 2018/2019 biennium, 68,220 new cases of cancer are expected in Brazil (66.12 new cases/100,000 inhabitants) [17], and it is believed that pain will remain one of the most frequent symptoms in these patients.

Therefore, it is necessary to evaluate nurses’ knowledge about cancer pain management, as well as to identify the factors associated with the knowledge of oncology nurses of a high complexity oncology centre (HCOC) in Brazil on the management of cancer pain, in order to identify the variables related to inadequate control and propose targeted educational interventions.

## Method

### Type of study, population and sample size

This is a cross-sectional study involving nurses working at a high complexity oncology reference centre in the city of Rio de Janeiro, Brazil. The study population was composed of 207 nurses. The study participants were selected through non-probabilistic sampling. The nurses were invited to participate in the research, through an invitation via institutional e-mail. The prevalence of knowledge in the management of cancer pain in studies ranged from 55% to 65% [13, 14, 18], however, the evaluation instrument was not the same as that used in the present study. The sample calculation of the present work assumed a level of confidence (α) of 5%, a power of 80% (1–β) and knowledge prevalence of 55%, computing 131 nurses, however, at the end of the study 126 nurses participated.

Data collection was performed between June and December 2015 by properly trained nurses, and the data collection instrument had already been tested in a study with nursing residents of the same institution [19], who were not part of the sample of the study. We attempted to carry out data collection in a quiet and private environment, close to the work environment of the professional, by the researchers involved.

The inclusion criteria were: to be a nurse, to work in inpatient units, and to be linked to the institution for at least one year. Exclusion criteria were professionals who were away for health or update reasons during the data collection period.

### Data collection instrument

The nurses were characterised in relation to sociodemographic and professional characteristics: gender, age, religion, training and professional experience, and general knowledge about pain. General knowledge about pain consisted of sources of information on cancer pain; approaches to pain; known therapeutic strategies; behaviour towards the patient with pain; and opinions about therapy with the use of opioid analgesics.

Knowledge about the management of cancer pain was evaluated through the instrument ‘Nurses’ Knowledge on Cancer Pain Management—WHO’. This tool was built following the recommendations of the WHO for the control of cancer pain. This assessment was submitted to content validation by experts, in the study developed by Ramos (1994) [20], corresponding to the only Brazilian instrument developed in order to evaluate knowledge about pain management in the cancer patient, and allows researchers to perform an assessment on the behaviour of nurses in relation to this population, regarding pain evaluation, control strategies and continuous care.

It is a self-report instrument, composed of 24 Likert-type items, distributed in three domains (pain evaluation, control strategies and continuous care), with 8 items each, and it has already been used in previous studies in Brazil [19, 21]. Each item is graded and scored according to the frequency of accomplishment of the statements that compose the instrument (always = 4.16 points, sometimes = 1.04 points and never = 0 points). The domain scores range from 0 to 33.28 points and the total score varies from 0 to 100 points and is obtained by adding the scores of each item [20] (Table 1).

In the present study, the reliability of the instrument was evaluated through internal consistency. The full scale (Cronbach’s α = 0.76) and the continuous care domain (Cronbach’s α = 0.74) showed good reliability. The pain evaluation (Cronbach’s α = 0.57) and control strategies domains (Cronbach’s α = 0.51) presented reliability considered as insufficient.

Knowledge about the management of cancer pain was analysed as a continuous and categorical variable. To establish the cutoffs per domain and for the total score, the receiver operating characteristic curve was used.

Assuming a 99.0% sensitivity and 1% false-negative ratio, the cut off point of the total score was 66.6 points (< 66.6 = inadequate knowledge; ≥ 66.6 = adequate knowledge); 22.4 points for the domains (< 22.4 = inadequate knowledge; ≥ 22.4 = adequate knowledge) pain assessment and continuous care and 22 points for the domain pain control strategies (< 22.0 = inadequate knowledge; ≥ 22.0 = adequate knowledge), results that are similar to those found in studies that used this instrument in nursing residents in the same institution [19, 21].

### Statistical analysis

Statistical analysis was developed in three stages: descriptive analysis, bivariate analysis, multiple analysis. All analyses were performed using R software, version 3.2.1.

The relationship between the classificatory variables was assessed using the Pearson chi-square test or Fisher’s exact test, and for quantitative variables the difference between the means was evaluated with the Student’s *t*-test or the Mann-Whitney U test, after verification of normality by the Shapiro-Wilk test.

In the multiple analysis, for the independent variables to knowledge about cancer pain management, the prevalence ratio (PR) and its respective confidence intervals (95% CI) were calculated using Poisson regression with robust variance, with adequate knowledge (yes/no) being the outcome. In these analyses, the sandwich library of the statistical R package, version 3.2.1, was used.

The independent variables were gender, age, professional training, length of professional experience, training time, position/performance and general knowledge about pain, information sources on cancer pain, theoretical knowledge about pain and analgesia, known therapeutic strategies and the opinion on therapy with the use of opioid analgesics.

The statistical modelling process was performed using the technique step by step, with the inclusion of independent variables one by one, in order to adjust the potentially confounding variables. The independent variables that presented a critical level of p ≤ 0.20 were considered candidates to remain in the final model. After the simultaneous inclusion of all main effects, plausible interactions were tested.

The final model selection considered the value of the Akaike information criterion, residue analysis by graphic observation and epidemiological significance. Values of *p* ≤ 0.05 were considered significant.

The study was approved by the Research Ethics Committee of the institution involved CAAE (Certificate of Presentation for Ethical Appreciation): 13594613.0.000.5374, according to guidelines and regulatory standards for research involving human beings, coming from Resolution 466 of 2012 of the National Health Council, and all participants signed the free informed consent term in two copies.

## Results

Most of the nurses were female (88.1%), aged between 30 and 49 years old (69.0%), graduated (96.0%), with more than 10 years of training (57, 9%) and professional experience (60.3%), and 82.5% were attending nurses (Table 2).

The prevalence of adequate knowledge was 54.1% (95% CI 45.40%–62.80%). Professionals with adequate knowledge were older, had longer professional training time and had more professional experience (*p* = 0.001) when compared to professionals with inadequate knowledge (Table 2).

In the total sample, approximately 25.0% of the nurses received information about cancer pain in the undergraduate programme; however, for most of them, this subject was addressed in professional practice (81.0%) and (52.4%) in the Graduate Programme. Almost all professionals reported to the known aspects related to pain assessment (92.1%) and only 18.3% reported to know about the theory of pain (Table 3). In addition, the most well-known therapeutic strategies were drug analgesia (99.2%) and adjuvant drugs (75.4%), and the least known were hypnosis (13.5%) and Do-In exercises (9.5%). Regarding opioid medications, the majority (52.4%) did not believe that opioids could cause problems for the patients and 73.8% of the nurses were against the use of placebos (Table 3).

Regarding nurses with inadequate knowledge about the management of cancer pain, nurses with adequate knowledge listed continuing education events as a source of information on cancer pain (36.8% versus 20.7%) in a higher frequency, and stood out in the knowledge of ethical issues (26.5% versus 12.1%) and neurolytic block of the coeliac plexus (39.7% versus 20.7%) as a therapeutic strategy for pain control (Table 3).

Nurses with adequate knowledge presented higher averages (*p* = 0.0001) in all domains of the instrument (pain evaluation, control strategy and continuous care), as well as in the total score when compared to those with inadequate knowledge (Table 4).

After the multiple analysis completion, professional experience time and not believing that the use of opioids causes harm to patients (Table 4) remained associated with adequate knowledge on cancer pain management. There was a progressive increase in the chance of adequate knowledge with the increase of professional experience time [10–19 years (PR = 1.72; 95% CI: 1.05–2.81); 20–29 years (PR = 2.56, 95% CI: 1.63–4.02); (PR = 3.45, 95% CI: 2.25–5.29)], and nurses who reported not believing that the use of opioids could cause harm to patients presented a greater chance (PR = 1.20; 95% CI: 1.12–2.00) to have adequate knowledge (Table 5).

## Discussion

The adequate control of pain depends on a multidisciplinary team. In this context, nursing professionals play a strategic role, being the professionals that most frequently assess the pain of the patients and report on the adverse effects of the therapy implemented. Thus, for the adequate management of pain in the cancer patient, it is necessary that the nurses perform the following actions: evaluate pain in a continuous and systematised way; play a leading role in its identification; to act as patient, family and professional educators regarding oncological pain, its pharmacological and non-pharmacological treatment; and as to demystify erroneous beliefs and thereby promote greater adherence to treatment [1, 13, 14].

The present study showed that nurses who worked in an HCOC in Brazil presented, in their majority, adequate knowledge regarding the management of cancer pain, according to the instrument ‘Nurses’ Knowledge on Cancer Pain Management—WHO ‘[20]. It is emphasised that this finding was superior to that observed in Brazilian studies that used the same evaluation instrument [20, 21], and lower than that observed in international studies [13, 14, 18].

Among Brazilian nurses attending cancer patients, in Salvador, Bahia, 40.2% had adequate knowledge on cancer pain management [20], while among nurses in the oncology residency programme area, the prevalence of adequate knowledge was only 31.8% [20].

Although in this study the majority of nurses were found to have adequate knowledge, the observed prevalence is below the expected, due to the fact that the nurses worked in a high-complexity oncology centre, with a high proportion of professionals with graduate-level training, in which content on cancer pain was studied (52.4%).

However, there was no significant difference between pain knowledge and professional training. This finding is similar to that observed in Brazilian nurses undergoing specialisation in oncology, using the same instrument of evaluation as the present study [19], which found that in Turkish and North American nurses, there was no statistically significant difference between the average of correct answers in the ‘knowledge and attitudes survey regarding pain (KASRP)’ and the level of education of nurses [26, 27]. However, it is contrary to the studies that show an increase in the average number of correct answers in the instruments for assessing the knowledge on pain management as the level of education of nurses increased, being higher in nurses with a Master’s degree [28, 29].

It is believed that the result observed in nurses in Brazil is due to the limited coverage of this subject in undergraduate courses, and when it occurs, it is carried out without the concern of interconnecting this content with previous knowledge, favouring the low prevalence of knowledge on the management of pain in newly trained nurses in the process of specialisation in oncology [19, 21].

Another possible explanation may be related to the fact that the nurses of the present study have mostly obtained their graduate-level degree in the modality of residency programmes, which are defined as in-service training; in other words, they prioritise the practice scenarios to the detriment of theoretical teaching, which in many cases comprises only 20% of the total course workload [19]. Therefore, graduate-level training was not associated with better knowledge of pain management in cancer patients [19, 21, 30, 31]. The findings of the present study corroborate the need of restructuring the specialisation format in a residency format, in order to have greater articulation between theory and practice, since theoretical basics support clinical practice [30, 31].

The results observed in nurses in Turkey show the importance of including courses on pain both in undergraduate and Master’s degree courses, and of the training promoted by the Ministry of Health on this subject, which may have increased nurses’ knowledge about this subject both of the new graduates and of the nurses who already worked in clinical practice. Thus, there was no difference in knowledge according to the degree of training of nurses [26]. Corroborating this hypothesis, a study developed in Taiwan showed that nurses who received previous training on pain had a higher mean of adequate knowledge in KASRP when compared to nurses who did not receive training (19.30 versus 17.06, *p* = 0.0001) [28], a similar result was verified in Korean nurses (*β* = 1.75, *p* ≤ 0.005) [14].

However, it is important to note that the source of education about pain management has an influence on the level of knowledge of nurses. Nurses who obtained education at conferences or professional training presented a higher average of correct answers when compared to those who received pain information in undergraduate courses (21.05 versus 17.06, *p* ≤ 0.05). Likewise, in the present study it was verified that nurses with adequate knowledge had a higher prevalence of information source on pain in update events (36.8% versus 20.7%, *p* = 0.04) when compared to those with inadequate knowledge [28].

It is necessary to observe that the studies developed in Brazil and the international studies used different instruments to qualify nurses’ knowledge on pain in oncology, which may have influenced the findings of these studies, so a comparison of these should be made with caution. For the instruments present disparities in the level of difficulty of the questions and show different cut-off points to characterise individuals with adequate or inadequate knowledge [13, 14, 22, 23].

In the Brazilian studies that evaluated pain through the questionnaire ‘Nurses’ Knowledge on Cancer Pain Management-WHO’ [20], the individuals with adequate knowledge were those that reached a percentage of 66% of correct answers, while in the majority of the international studies on the subject, in which the instrument used was KASRP, the cutoff points were higher (80.0% of correct answers) [22, 24–26].

Also, the level of difficulty of the instruments presents important differences. In the instrument ‘Nurses’ Knowledge on Cancer Pain Management-WHO’, the individual scores in two response categories (always = 4.16 points and sometimes = 1.04 points). On the other hand, in the KASRP the items are categorised and scored as correct (1) and incorrect (0), and therefore, the individual only has a punctuation possibility [32].

In spite of the differences between these two instruments, in the present study the instrument used was sensitive to capturing differences among nurses with adequate and inadequate knowledge regarding the management of cancer pain. Considering that nurses with adequate knowledge had a higher average in the total score and in all domains of the instrument, and noticing that the difference of knowledge between the groups was statistically significant, this corroborates the findings of studies that used this instrument in nurses in the process of training at the graduate level in oncology [19, 21].

In addition, it was observed that nurses classified with adequate knowledge showed a better understanding about the ethical aspects related to cancer pain, alternative measures for pain control (coeliac plexus neuroleptic block) when compared to nurses with inadequate knowledge. However, the prevalence of previous knowledge about these subjects is still very small, especially because they are nurses who deal daily with cancer patients, and it is known that pain is one of the most prevalent symptoms in this clientele [2].

Also, we note the low prevalence of nurses who reported previous knowledge regarding pain theory, type of pain, and non-pharmacological strategies to control pain. We also note the fact that 26.7% of nurses are in favour of the use of placebos in treatment, a strategy that is considered unethical [33], and that 47.6% of them believe that opioids do harm to patients. It was expected that nurses who work exclusively in the care of people living with cancer in a High Complexity Centre would present greater knowledge about these themes; however, our findings are similar to those that have been evidenced in Brazilian studies since the 1990s [34].

These dysfunctional beliefs are associated with the lack of knowledge of themes of extreme importance for the understanding and adequate management of pain. These beliefs may contribute to the persistence of pain in about 43% of cancer patients [3], even given the presence of a medical prescription and the availability of analgesics [2, 3, 18]. We must consider that, although the nursing team is not responsible for the prescription of medication, they are still in charge of identifying the pain and deciding on the prescribed analgesic medication, especially those that are established as administration if necessary [13, 14].

Still, regarding the factors associated with knowledge about the management of cancer pain in nurses, in the present study it was evidenced in the multiple model that adequate knowledge about the management of cancer pain was dependent on the amount of professional experience and beliefs or knowledge related to the use of opioids.

The amount of professional experience is directly related to the opportunities of contact with the subject, through reading, updating or graduate courses, continuing education activities, and participation in related technical-scientific activities. Still, the longer clinical practice may contribute to knowledge about pain management, since professionals with more professional experience time may have had greater contact with clinical situations related to pain and analgesia. Therefore, the results of our study are in line with the results observed in Brazilian nurses in the process of specialisation in oncology [19], North American [27] and Korean nurses [28], since the professionals with more time of professional practice have gained better knowledge about painful phenomena. However, it was different from the findings by Wilson (2007) [35], in which nurses with a specialisation in oncology presented greater expertise regarding pain management when compared to generalist nurses; however, this knowledge was not associated with professional experience.

Adequate knowledge about pain management was also dependent on the non-occurrence of dysfunctional beliefs about opioid use. In that regard, a study conducted with Iranian nurses showed a negative correlation between adequate knowledge and perception of threat from the treatment proposed for the patient [36]. These beliefs still constitute one of the main barriers observed between patients [14] and professionals [7, 14, 37], for adequate pain control, involving prejudice and fear of side effects, tolerance and dependence [2].

Dysfunctional beliefs about opioid use may be associated with a lack of knowledge regarding pain theory, pharmacokinetics, and pharmacodynamics of this class of medications, contributing to many nurses being afraid to administer these medications due to fear of psychological dependence and respiratory depression, which has been amply demonstrated in several studies [7, 13, 14, 34, 36].

It is known that dysfunctional beliefs limit adequate pain management by promoting resistance to change. Considering the data related to the inadequate control of cancer pain and the high prevalence of pain in people living with cancer, it is necessary to reflect on the factors that may influence the appropriate management of pain. In this sense, some questions do arise, but they are beyond the scope of the study. Is it likely that professional experience, combined with adequate knowledge—and possibly the routine of administering opioids and managing their effects—can modulate cultural barriers and negative attitudes related to cancer pain? Furthermore, do these nurses feel more comfortable regarding the management of opioids due to the experience with patients who present intense pain syndromes associated with the existence of a consistent institutional analgesia protocol?

To answer these questions, qualitative studies to explore the phenomenon among professional groups are necessary. Therefore, the present study aims to contribute to the raising of these research questions for future work, adding more knowledge on the barriers involved in the management of cancer pain.

The limitation of this study is related to the instrument used to evaluate the knowledge of cancer pain management [20]. At at the time of its construction, this tool was submitted to content validation by experts, but, had not yet had its psychometric validity and reliability properties well-established, which should be proposed by future studies.

However, it is emphasised that in this study, the instrument showed good reliability in relation to the total score (α Cronbach = 0.76) and discriminated between individuals with adequate knowledge and those with inadequate knowledge in all domains of the instrument; and in the total score, which shows its validity as construct, the same was observed in studies carried out with nurses in the oncology training process [19, 21].

The present study contributed to indicate the prevalence of adequate knowledge on the management of cancer pain among nurses at a hospital specialised in cancer treatment, and the association of this knowledge with sociodemographic and professional variables, and previous knowledge about pain.

## Conclusion

The present study identified that the majority of nurses presented adequate knowledge about the management of cancer pain. These nurses presented higher average scores in all domains of the instrument when compared to those with inadequate knowledge. A difference was observed between these nurses and their sources of acquiring knowledge about pain, knowledge of alternative methods for pain control (coeliac plexus block), longer training time, and professional experience. Moreover, the professional experience and knowledge or beliefs related to the use of opioid analgesics remained associated with adequate knowledge on the management of cancer pain in the multiple model. Given these findings, actions of continuing education, updating education and reflection are imperative, particularly for professionals with less professional experience.

## Conflicts of interest

The authors declare that there are no conflicts of interest.

## Figures and Tables

**Table 1.** ‘Nurses’ knowledge of cancer pain management - WHO’. This instrument was built following the recommendations of the WHO for the control of cancer pain, developed by Ramos (1994).

Distribution of items/pain assessment
**Items/Domains**
1. Believes in patient complaints
2. Measures the intensity of pain
3. Notes the patient’s psychological state
4. Performs physical examination
5. Considers alternative methods of pain control in the initial evaluation
6. Assesses the level of pain control after starting treatment
7. Reviews the diagnosis in case of doubt of the cause of the pain
8. Makes a detailed history of the pain complaint
**Control strategies**
1. Adapts the strategies to individual needs
2. Performs therapeutic measures to minimise undesirable effects
3. Develops alternative strategies for pain control
4. Believes that the dose of medication should be individual
5. Believes in the use of oral medication as a preferred administration method for pain relief
6. Promotes strategies to increase sleep hours and fight insomnia
7. Believes the dose of medication promotes pain relief by four hours
8. Administers medication for cancer pain, even without patient´s complaint
**Continuous care**
1. Seeks to ensure biopsychosocial well-being
2. Develops actions to relieve other symptoms resulting from the disease
3. Establishes a support system for the patient to live actively
4. Controls side effects of drug usage
5. Thinks it is important to include the family in the care of the patient with pain
6. Includes family and friends in home care training
7. Promotes strategies so that the patient is not socially alone
8. Promotes strategies so that the terminal patient does not suffer pain

**Table 2.** Sociodemographic and professional characteristics of nurses, according to their knowledge status on the management of cancer pain (*n* = 126). Rio de Janeiro, RJ, Brazil, 2018.

Variables	Adequate knowledge	Inadequate knowledge	Total	*P* value
	*n*(%)	*n*(%)	*n*(%)	
Gender				
Male	8 (11.7)	7 (12.1)	15 (11.9)	0.580
Female	60 (88.2)	51 (87.9)	111 (88.1)	
Age group				
20–29	5 (7.4)	14 (24.1)	19 (15.1)	0.001
30–39	21 (31.0)	28 (48.3)	49 (38.9)	
40–49	26 (38.2)	12 (20.7)	38 (30.2)	
50–59	16 (23.5)	4 (7.0)	20 (15.9)	
Professional qualification				
Undergraduate	3 (4.5)	2 (3.4)	5 (4.0)	0.620
Graduate	64 (95.5)	56 (96.6)	121 (96.0)	
Training time				
<10 years	18 (26.4)	35 (60.3)	53 (42.1)	0.001
10–19 years	20 (29.4)	16 (28.0)	36 (28.6)	
20–29 years	20 (29.4)	7 (12.1)	27 (21.4)	
30–39 years	10 (14.8)	0 (0.0)	10 (7.9)	
Time of professional experience				
<10 years	16 (23.5)	34(58.6)	50 (39.7)	0.001
10–19 years	19 (28.0)	18 (31.0)	37 (29.4)	
20–29 years	20 (29.4)	6 (10.3)	26 (20.6)	
30–39 years	13 (19.1)	0	13 (10.3)	
Position/Professional Practise				
Head of service/Head of unit/Supervision	14 (20.6)	8 (13.8)	22 (17.5)	0.230*
Attending nurse	54 (79.4)	50 (86.2)	104 (82.5)	

* : *Fischer’s exact test*

**Table 3.** Previous knowledge of nurses about pain according to the knowledge status on the management of cancer pain (*n* = 126). Rio de Janeiro, RJ, Brazil, 2018

Variables	Adequate knowledge	Inadequate knowledge	Total	P value
	*n*(%)	*n*(%)	*n*(%)	
Sources of cancer pain information				
Undergraduation programme	16 (23.5)	15 (25.9)	31 (24.6)	0.760
Graduation programme	35 (51.5)	25 (43.1)	66 (52.4)	0.350
In-service training	30 (51.7)	22 (37.9)	52 (41.3)	0.480
Update on events	25 (36.8)	12 (20.7)	37 (29.4)	0.040
Professional practice	10 (14.7)	14 (24.1)	102 (81.0)	0.180
Theoretical knowledge of pain and analgesia				
Pain Physiology	50 (73.5)	37 (63.8)	87 (69.0)	0.240
Pain types	52 (76.5)	43 (74.1)	93 (73.8)	0.280
Ethical aspects	18 (26.5)	7 (12.1)	25 (19.8)	0.040
Behavioural aspects	40 (58.8)	41 (70.7)	45 (35.7)	0.120
Drug treatment of pain	59 (86.9)	50 (86.2)	109 (86.5)	0.910
Non-pharmacological measures of pain relief	49 (72.1)	39 (67.2)	88 (69.8)	0.560
Theory of pain	15 (22.1)	8 (13.8)	23 (18.3)	0.230
Pain assessment	64 (94.1)	52 (89.7)	116 (92.1)	0.360
Cultural aspects	15 (22.1)	10 (17.2)	25 (19.8)	0.320
Known therapeutic strategies				
Analgesia medication	67 (98.5)	58 (100)	125 (99.2)	0.350
Adjuvant Medications for Pain Relief	55 (80.9)	40 (69.0)	95 (75.4)	0.120
Alternative measures for pain relief	45 (66.2)	38 (65.5)	83 (65.9)	0.540
Occupational therapy	23 (33.8)	14(24.1)	37 (29.4)	0.230
Do-In	8 (11.8)	4 (6.9)	12 (9.5)	0.350*
Hypnosis	11 (16.2)	6 (10.3)	17 (13.5)	0.340*
Acupuncture	19 (27.9)	18 (31.0)	89 (70.6)	0.700
Cordotomy	10 (14.7)	6(10.3)	16 (12.7)	0.470*
Neurolytic block of the coeliac plexus	27 (39.7)	12 (20.7)	39 (31.0)	0.020
Transcutaneous neurostimulation	19 (27.9)	8 (13.7)	27 (21.4)	0.060
Do you believe that opioids do wrong to patients?	37 (54.4)	23 (39.7)	60 (47.6)	0.090
Regarding the use of placebos for patients with cancer pain are you?				
For	19 (27.9)	14 (24.1)	33 (26.2)	0.630
Against	49 (72.1)	44 (75.9)	93 (73.8)	

* : *Fischer’s exact test*

**Table 4.** Comparison of domain scores on pain assessment, control strategies and continuous care, according to the status of knowledge on the management of cancer pain (*n* = 126). Rio de Janeiro, RJ, Brazil, 2018.

Knowledge about pain management	Instrument Domains							
	Pain assessment (%)	*P* value	Control Strategies (%)	*P* value	Continuous care (%)	*P* value	Total (%)	*P* value *
Adequate	59.2	0.0001	23.8	0.0001	70.6	0.0001	54	0.0001
Mean (SD)	26.6 (3.6)		21.3 (4.3)		28.7 (4.0)		76.6 (7.4)	
Inadequate	40.8		76.2		29.4		46	
Mean (SD)	19.5 (4.4)		16.0 (4.0)		20.6 (6.4)		56.2 (7.1)	
Total	100		100		100		100	
Mean (SD)	23.33 (5.3)		18.9 (5.0)		25.0 (6.6)		67.2 (12.6)	

* : Mann-Whitney U test

**Table 5.** Variables associated with adequate knowledge about the management in cancer pain (n = 126). Rio de Janeiro, RJ, Brazil, 2018.

Variables		Adequate knowledge		
Time of professional experience	*n*	%	*PR not adjusted (CI95%)	*PR adjusted (CI95%)
<10 years	50	39.7	1	
10–19 years	37	29.4	1.60 (0.96–2.70)	1.72 (1.05–2.81)
20–29 years	26	20.6	2.43 (1.54–3.81)	2.56 (1.63–4.02)
30–39 years	13	10.3	3.12 (2.09–4.87)	3.45 (2.25–5.29)
Believes that the use of opioids does harm to patients				
No	66	52.4	1.31 (0.95–1.82)	1.20 (1.12–2.00)
Yes	60	47.6	1	

* PR: Prevalence ratio
